# (2,6-Difluoro­phen­yl)(4-methyl­piperidin-1-yl)methanone

**DOI:** 10.1107/S1600536811033848

**Published:** 2011-08-27

**Authors:** Mohammad T. M. Al-Dajani, Hassan H. Adballah, Nornisah Mohamed, Madhukar Hemamalini, Hoong-Kun Fun

**Affiliations:** a School of Pharmaceutical Sciences, Universiti Sains Malaysia, 11800 USM, Penang, Malaysia; b School of Chemical Sciences, Universiti Sains Malaysia, 11800 USM, Penang, Malaysia; c X-ray Crystallography Unit, School of Physics, Universiti Sains Malaysia, 11800 USM, Penang, Malaysia

## Abstract

In the title compound, C_13_H_15_F_2_NO, the piperidine ring adopts a chair conformation. The dihedral angle between the least-squares plane of the piperidine ring and the benzene ring is 48.75 (7)°. In the crystal structure, the mol­ecules are connected *via* C—H⋯O hydrogen bonds, forming a zigzag chain along the *b* axis.

## Related literature

For the biological applications of piperidine derivatives, see: Waelbroeck *et al.* (1992 ▶); El Hadri *et al.* (1995 ▶). For puckering parameters, see: Cremer & Pople (1975 ▶).
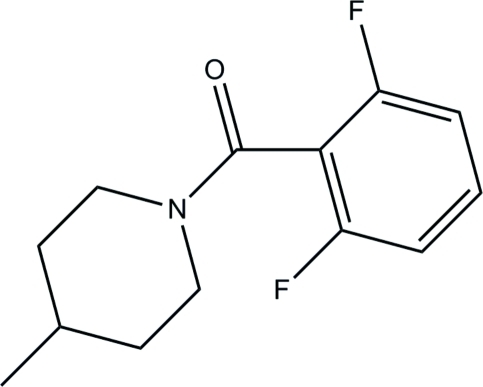


## Experimental

### 

#### Crystal data

C_13_H_15_F_2_NO
*M* *_r_* = 239.26
Monoclinic, 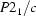

*a* = 9.1807 (7) Å
*b* = 10.9910 (8) Å
*c* = 13.2477 (8) Å
β = 115.582 (4)°
*V* = 1205.71 (15) Å^3^
*Z* = 4
Mo *K*α radiation
μ = 0.10 mm^−1^
*T* = 296 K
0.43 × 0.38 × 0.19 mm

#### Data collection

Bruker APEXII DUO CCD area-detector diffractometer
Absorption correction: multi-scan (*SADABS*; Bruker, 2009 ▶) *T*_min_ = 0.956, *T*_max_ = 0.981
11030 measured reflections
3513 independent reflections
2617 reflections with *I* > 2σ(*I*)
*R*_int_ = 0.018

#### Refinement

*R*[*F* ^2^ > 2σ(*F* ^2^)] = 0.045
*wR*(*F* ^2^) = 0.136
*S* = 1.06
3513 reflections
155 parameters
H-atom parameters constrained
Δρ_max_ = 0.23 e Å^−3^
Δρ_min_ = −0.20 e Å^−3^

### 

Data collection: *APEX2* (Bruker, 2009 ▶); cell refinement: *SAINT* (Bruker, 2009 ▶); data reduction: *SAINT*; program(s) used to solve structure: *SHELXTL* (Sheldrick, 2008 ▶); program(s) used to refine structure: *SHELXTL*; molecular graphics: *SHELXTL*; software used to prepare material for publication: *SHELXTL* and *PLATON* (Spek, 2009 ▶).

## Supplementary Material

Crystal structure: contains datablock(s) global, I. DOI: 10.1107/S1600536811033848/is2763sup1.cif

Structure factors: contains datablock(s) I. DOI: 10.1107/S1600536811033848/is2763Isup2.hkl

Supplementary material file. DOI: 10.1107/S1600536811033848/is2763Isup3.cml

Additional supplementary materials:  crystallographic information; 3D view; checkCIF report

## Figures and Tables

**Table 1:** Hydrogen-bond geometry (Å, °)

*D*—H⋯*A*	*D*—H	H⋯*A*	*D*⋯*A*	*D*—H⋯*A*
C3—H3*A*⋯O1^i^	0.93	2.35	3.2646 (18)	168

Symmetry code: (i) 
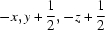
.

**Fig. 1.**
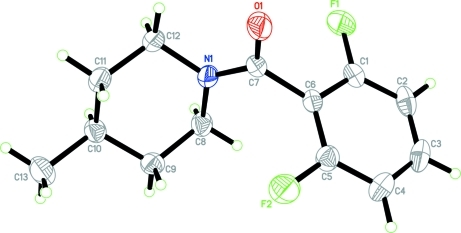
The molecular structure of the title compound, showing 50% probability displacement ellipsoids and the atom-numbering scheme.

**Fig. 2.**
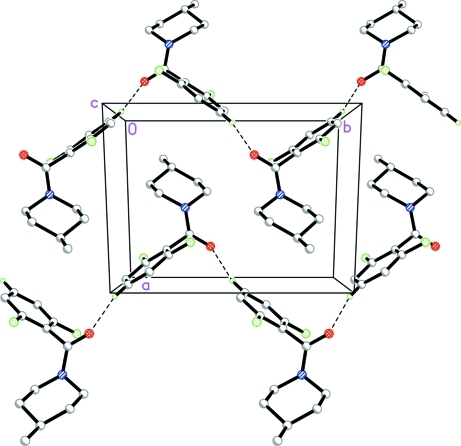
The crystal packing of the title compound, viewed along the c axis. Dashed lines represent C—H···O hydrogen bonds.

## supplementary crystallographic information

### Comment

The piperidine nucleus is present in a wide range of biologically active compounds. For example, the binding properties of 4-diphenyl acetoxy-*N*-methylpiperidine methiodide (4-DAMP) and its analogs have been evaluated on muscarinic receptors in human neuroblastoma NB-OK1 cells (M1 receptor subtype), rat heart (M2 subtype), rat pancreas (M3 subtype) and the putative M4 receptor subtype in striatum (Waelbroeck *et al.*, 1992). NMDA receptor antagonist properties of piperidine-2-carboxylic acid derivatives have also been reported (El Hadri *et al.*, 1995).  Due to their biological importance of piperidine derivatives, herein, we have present the crystal structure of the title compound (I).

The molecular structure of the title compound is shown in Fig. 1. The piperidine (N1/C8–C12) ring adopts a chair conformation [puckering parameters: Q = 0.5569 (14) Å, θ = 2.24 (14)° and φ = 132 (4)° (Cremer & Pople, 1975)] with atoms C8 and C10 deviating by 0.230 (1) and 0.238 (1) Å from the least-squares plane defined by the remaining atoms (N1/C9/C11–C12) in the ring.  The dihedral angle between the least-squares plane of the piperidine (N1/C8–C12) ring and the fluoro-subsituted benzene (C1–C6) ring is 48.75 (7)°.

In the crystal structure, the molecules are connected *via* C—H···O hydrogen bonds (Table 1) forming one-dimensional supramolecular chains along the *b* axis (Fig. 2).

### Experimental

In a round bottom flask, 25ml of toluene was mixed with 4-methylpiperidine (0.01 mol, 1.0 g) with stirring.  Drops of 2,6-difluorobenzylchloride (0.01 mol, 1.7g) dissolved in toluene was then added.  The reaction mixture was refluxed for 30 min. The yellow precipitate formed was washed with chloroform and with water. The precipitate was then dissolved in methanol at room temperature. After few days, colourless needle-shaped crystals were formed by slow evaporation.

### Refinement

All H atoms were positioned geometrically (C—H = 0.93–0.97 Å) and were refined using a riding model, with *U*_iso_(H) = 1.2 or 1.5*U*_eq_(C).  A rotating group model was used for the methyl group.

### Crystal data

**Table:**

C_13_H_15_F_2_NO	*F*(000) = 504
*M**_r_* = 239.26	*D*_x_ = 1.318 Mg m^−^^3^
Monoclinic, *P*2_1_/*c*	Mo *K*α radiation, λ = 0.71073 Å
Hall symbol: -P 2ybc	Cell parameters from 4002 reflections
*a* = 9.1807 (7) Å	θ = 2.5–29.6°
*b* = 10.9910 (8) Å	µ = 0.10 mm^−^^1^
*c* = 13.2477 (8) Å	*T* = 296 K
β = 115.582 (4)°	Block, colourless
*V* = 1205.71 (15) Å^3^	0.43 × 0.38 × 0.19 mm
*Z* = 4	

### Data collection

**Table:**

Bruker APEXII DUO CCD area-detector diffractometer	3513 independent reflections
Radiation source: fine-focus sealed tube	2617 reflections with *I* > 2σ(*I*)
graphite	*R*_int_ = 0.018
φ and ω scans	θ_max_ = 30.1°, θ_min_ = 2.5°
Absorption correction: multi-scan (*SADABS*; Bruker, 2009)	*h* = −12→12
*T*_min_ = 0.956, *T*_max_ = 0.981	*k* = −13→15
11030 measured reflections	*l* = −18→18

### Refinement

**Table:**

Refinement on *F*^2^	Primary atom site location: structure-invariant direct methods
Least-squares matrix: full	Secondary atom site location: difference Fourier map
*R*[*F*^2^ > 2σ(*F*^2^)] = 0.045	Hydrogen site location: inferred from neighbouring sites
*wR*(*F*^2^) = 0.136	H-atom parameters constrained
*S* = 1.06	*w* = 1/[σ^2^(*F*_o_^2^) + (0.0678*P*)^2^ + 0.1308*P*] where *P* = (*F*_o_^2^ + 2*F*_c_^2^)/3
3513 reflections	(Δ/σ)_max_ = 0.001
155 parameters	Δρ_max_ = 0.23 e Å^−^^3^
0 restraints	Δρ_min_ = −0.20 e Å^−^^3^

### Special details

**Table:**

Geometry. All s.u.'s (except the s.u. in the dihedral angle between two l.s. planes) are estimated using the full covariance matrix. The cell s.u.'s are taken into account individually in the estimation of s.u.'s in distances, angles and torsion angles; correlations between s.u.'s in cell parameters are only used when they are defined by crystal symmetry. An approximate (isotropic) treatment of cell s.u.'s is used for estimating s.u.'s involving l.s. planes.
Refinement. Refinement of F^2^ against ALL reflections. The weighted R-factor wR and goodness of fit S are based on F^2^, conventional R-factors R are based on F, with F set to zero for negative F^2^. The threshold expression of F^2^ > 2σ(F^2^) is used only for calculating R-factors(gt) etc. and is not relevant to the choice of reflections for refinement. R-factors based on F^2^ are statistically about twice as large as those based on F, and R- factors based on ALL data will be even larger.

### Fractional atomic coordinates and isotropic or equivalent isotropic displacement parameters (Å^2^)

**Table:**

	*x*	*y*	*z*	*U*_iso_*/*U*_eq_	
F1	0.26788 (12)	0.67786 (9)	0.26565 (7)	0.0733 (3)	
F2	0.16103 (13)	0.89859 (9)	0.52606 (7)	0.0835 (3)	
O1	0.23349 (12)	0.59626 (9)	0.48443 (8)	0.0697 (3)	
N1	0.46198 (12)	0.70862 (9)	0.55150 (9)	0.0528 (3)	
C1	0.19420 (13)	0.77289 (11)	0.28922 (9)	0.0476 (3)	
C2	0.10112 (15)	0.85157 (14)	0.20507 (10)	0.0600 (4)	
H2A	0.0896	0.8407	0.1324	0.072*	
C3	0.02604 (15)	0.94608 (15)	0.23065 (12)	0.0638 (4)	
H3A	−0.0384	0.9992	0.1744	0.077*	
C4	0.04459 (16)	0.96375 (14)	0.33859 (12)	0.0625 (4)	
H4A	−0.0058	1.0283	0.3562	0.075*	
C5	0.13981 (15)	0.88297 (12)	0.41937 (10)	0.0517 (3)	
C6	0.21693 (12)	0.78571 (10)	0.39862 (8)	0.0413 (2)	
C7	0.30603 (14)	0.68901 (10)	0.48395 (9)	0.0455 (3)	
C8	0.54606 (14)	0.82462 (12)	0.56603 (10)	0.0526 (3)	
H8A	0.4746	0.8839	0.5139	0.063*	
H8B	0.6391	0.8145	0.5502	0.063*	
C9	0.60040 (14)	0.86998 (11)	0.68488 (10)	0.0504 (3)	
H9A	0.5064	0.8871	0.6981	0.060*	
H9B	0.6604	0.9451	0.6946	0.060*	
C10	0.70612 (14)	0.77668 (11)	0.76970 (10)	0.0503 (3)	
H10A	0.8025	0.7636	0.7570	0.060*	
C11	0.61572 (15)	0.65643 (11)	0.74973 (11)	0.0563 (3)	
H11A	0.6864	0.5953	0.7997	0.068*	
H11B	0.5238	0.6660	0.7670	0.068*	
C12	0.55722 (17)	0.61347 (11)	0.62987 (11)	0.0631 (4)	
H12A	0.6493	0.5925	0.6156	0.076*	
H12B	0.4915	0.5411	0.6185	0.076*	
C13	0.7612 (2)	0.82022 (17)	0.88950 (12)	0.0781 (5)	
H13A	0.8241	0.8931	0.9009	0.117*	
H13B	0.8258	0.7583	0.9402	0.117*	
H13C	0.6686	0.8368	0.9031	0.117*	

### Atomic displacement parameters (Å^2^)

**Table:**

	*U*^11^	*U*^22^	*U*^33^	*U*^12^	*U*^13^	*U*^23^
F1	0.0883 (6)	0.0761 (6)	0.0598 (5)	0.0042 (5)	0.0360 (4)	−0.0189 (4)
F2	0.1137 (8)	0.0920 (7)	0.0538 (5)	0.0326 (6)	0.0448 (5)	0.0010 (4)
O1	0.0689 (6)	0.0515 (5)	0.0607 (6)	−0.0175 (4)	0.0017 (4)	0.0058 (4)
N1	0.0477 (5)	0.0406 (5)	0.0503 (5)	0.0007 (4)	0.0025 (4)	0.0013 (4)
C1	0.0445 (5)	0.0567 (7)	0.0384 (5)	−0.0074 (5)	0.0147 (4)	−0.0065 (4)
C2	0.0545 (7)	0.0820 (9)	0.0331 (5)	−0.0143 (6)	0.0091 (5)	0.0061 (5)
C3	0.0429 (6)	0.0752 (9)	0.0575 (7)	0.0000 (6)	0.0069 (5)	0.0263 (7)
C4	0.0511 (6)	0.0641 (8)	0.0702 (8)	0.0153 (6)	0.0243 (6)	0.0146 (6)
C5	0.0520 (6)	0.0601 (7)	0.0439 (6)	0.0077 (5)	0.0214 (5)	0.0032 (5)
C6	0.0380 (5)	0.0456 (5)	0.0350 (5)	−0.0021 (4)	0.0107 (4)	−0.0002 (4)
C7	0.0495 (6)	0.0409 (5)	0.0364 (5)	−0.0022 (4)	0.0093 (4)	−0.0035 (4)
C8	0.0447 (5)	0.0528 (7)	0.0501 (6)	−0.0060 (5)	0.0110 (5)	0.0037 (5)
C9	0.0463 (6)	0.0418 (6)	0.0578 (7)	−0.0074 (5)	0.0175 (5)	−0.0042 (5)
C10	0.0443 (5)	0.0553 (7)	0.0446 (6)	−0.0016 (5)	0.0130 (4)	−0.0039 (5)
C11	0.0523 (6)	0.0473 (6)	0.0547 (7)	0.0042 (5)	0.0094 (5)	0.0074 (5)
C12	0.0613 (7)	0.0417 (6)	0.0580 (7)	0.0084 (5)	−0.0009 (6)	−0.0013 (5)
C13	0.0974 (12)	0.0762 (10)	0.0507 (8)	−0.0106 (9)	0.0224 (8)	−0.0110 (7)

### Geometric parameters (Å, °)

**Table:**

F1—C1	1.3522 (15)	C8—H8A	0.9700
F2—C5	1.3518 (14)	C8—H8B	0.9700
O1—C7	1.2192 (15)	C9—C10	1.5213 (17)
N1—C7	1.3381 (14)	C9—H9A	0.9700
N1—C8	1.4595 (16)	C9—H9B	0.9700
N1—C12	1.4671 (15)	C10—C13	1.5200 (18)
C1—C2	1.3777 (18)	C10—C11	1.5218 (18)
C1—C6	1.3795 (15)	C10—H10A	0.9800
C2—C3	1.368 (2)	C11—C12	1.5153 (19)
C2—H2A	0.9300	C11—H11A	0.9700
C3—C4	1.379 (2)	C11—H11B	0.9700
C3—H3A	0.9300	C12—H12A	0.9700
C4—C5	1.3747 (17)	C12—H12B	0.9700
C4—H4A	0.9300	C13—H13A	0.9600
C5—C6	1.3739 (17)	C13—H13B	0.9600
C6—C7	1.5094 (15)	C13—H13C	0.9600
C8—C9	1.5159 (17)		
			
C7—N1—C8	125.53 (10)	C8—C9—C10	111.36 (10)
C7—N1—C12	119.81 (10)	C8—C9—H9A	109.4
C8—N1—C12	114.19 (9)	C10—C9—H9A	109.4
F1—C1—C2	119.69 (11)	C8—C9—H9B	109.4
F1—C1—C6	117.24 (11)	C10—C9—H9B	109.4
C2—C1—C6	123.06 (12)	H9A—C9—H9B	108.0
C3—C2—C1	118.63 (12)	C13—C10—C9	112.18 (12)
C3—C2—H2A	120.7	C13—C10—C11	111.45 (12)
C1—C2—H2A	120.7	C9—C10—C11	109.32 (9)
C2—C3—C4	120.94 (12)	C13—C10—H10A	107.9
C2—C3—H3A	119.5	C9—C10—H10A	107.9
C4—C3—H3A	119.5	C11—C10—H10A	107.9
C5—C4—C3	117.94 (13)	C12—C11—C10	111.88 (12)
C5—C4—H4A	121.0	C12—C11—H11A	109.2
C3—C4—H4A	121.0	C10—C11—H11A	109.2
F2—C5—C6	117.00 (10)	C12—C11—H11B	109.2
F2—C5—C4	119.21 (12)	C10—C11—H11B	109.2
C6—C5—C4	123.79 (12)	H11A—C11—H11B	107.9
C5—C6—C1	115.62 (10)	N1—C12—C11	110.65 (10)
C5—C6—C7	123.88 (10)	N1—C12—H12A	109.5
C1—C6—C7	120.14 (10)	C11—C12—H12A	109.5
O1—C7—N1	124.08 (11)	N1—C12—H12B	109.5
O1—C7—C6	118.17 (10)	C11—C12—H12B	109.5
N1—C7—C6	117.69 (10)	H12A—C12—H12B	108.1
N1—C8—C9	110.03 (10)	C10—C13—H13A	109.5
N1—C8—H8A	109.7	C10—C13—H13B	109.5
C9—C8—H8A	109.7	H13A—C13—H13B	109.5
N1—C8—H8B	109.7	C10—C13—H13C	109.5
C9—C8—H8B	109.7	H13A—C13—H13C	109.5
H8A—C8—H8B	108.2	H13B—C13—H13C	109.5
			
F1—C1—C2—C3	179.14 (11)	C8—N1—C7—C6	13.13 (19)
C6—C1—C2—C3	−0.79 (19)	C12—N1—C7—C6	−175.26 (11)
C1—C2—C3—C4	0.9 (2)	C5—C6—C7—O1	94.43 (15)
C2—C3—C4—C5	−0.5 (2)	C1—C6—C7—O1	−78.49 (15)
C3—C4—C5—F2	179.55 (13)	C5—C6—C7—N1	−88.19 (15)
C3—C4—C5—C6	−0.2 (2)	C1—C6—C7—N1	98.90 (13)
F2—C5—C6—C1	−179.40 (11)	C7—N1—C8—C9	115.22 (13)
C4—C5—C6—C1	0.35 (19)	C12—N1—C8—C9	−56.81 (15)
F2—C5—C6—C7	7.40 (18)	N1—C8—C9—C10	56.43 (13)
C4—C5—C6—C7	−172.86 (12)	C8—C9—C10—C13	−179.72 (12)
F1—C1—C6—C5	−179.77 (10)	C8—C9—C10—C11	−55.56 (14)
C2—C1—C6—C5	0.16 (17)	C13—C10—C11—C12	178.83 (12)
F1—C1—C6—C7	−6.30 (16)	C9—C10—C11—C12	54.25 (14)
C2—C1—C6—C7	173.64 (11)	C7—N1—C12—C11	−116.98 (13)
C8—N1—C7—O1	−169.65 (13)	C8—N1—C12—C11	55.54 (17)
C12—N1—C7—O1	2.0 (2)	C10—C11—C12—N1	−53.71 (16)

### Hydrogen-bond geometry (Å, °)

**Table:**

*D*—H···*A*	*D*—H	H···*A*	*D*···*A*	*D*—H···*A*
C3—H3A···O1^i^	0.93	2.35	3.2646 (18)	168.

Symmetry codes: (i) −*x*, *y*+1/2, −*z*+1/2.
